# BioEve Search: A Novel Framework to Facilitate Interactive Literature Search

**DOI:** 10.1155/2012/509126

**Published:** 2012-05-28

**Authors:** Syed Toufeeq Ahmed, Hasan Davulcu, Sukru Tikves, Radhika Nair, Zhongming Zhao

**Affiliations:** ^1^Department of Biomedical Informatics, Vanderbilt University, Nashville, TN 37232, USA; ^2^Department of Computer Science and Engineering, Arizona State University, Tempe, AZ 85281, USA; ^3^Department of Cancer Biology, Vanderbilt University School of Medicine, Nashville, TN 37232, USA

## Abstract

*Background*. Recent advances in computational and biological methods in last two decades have remarkably changed the scale of biomedical research and with it began the unprecedented growth in both the production of biomedical data and amount of published literature discussing it. An automated extraction system coupled with a cognitive search and navigation service over these document collections would not only save time and effort, but also pave the way to discover hitherto unknown information implicitly conveyed in the texts. *Results*. We developed a novel framework (named “BioEve”) that seamlessly integrates Faceted Search (Information Retrieval) with Information Extraction module to provide an interactive search experience for the researchers in life sciences. It enables guided step-by-step search query refinement, by suggesting concepts and entities (like genes, drugs, and diseases) to quickly filter and modify search direction, and thereby facilitating an enriched paradigm where user can discover related concepts and keywords to search while information seeking. *Conclusions*. The BioEve Search framework makes it easier to enable scalable interactive search over large collection of textual articles and to discover knowledge hidden in thousands of biomedical literature articles with ease.

## 1. Background

Human genome sequencing marked the beginning of the era of large-scale genomics and proteomics, leading to large quantities of information on sequences, genes, interactions, and their annotations. In the same way that the capability to analyze data increases, the output by high-throughput techniques generates more information available for testing hypotheses and stimulating novel ones. Many experimental findings are reported in the -omics literature, where researchers have access to more than 20 million publications, with up to 4,500 new ones per day, available through to the widely used PubMed citation index and Google Scholar. This vast increase in available information demands novel strategies to help researchers to keep up to date with recent developments, as *ad hoc* querying with Boolean queries is tedious and often misses important information. 

Even though PubMed provides an advanced keyword search and offers useful query expansion, it returns hundreds or thousands of articles as result; these are sorted by publication date, without providing much help in selecting or drilling down to those few articles that are most relevant regarding the user's actual question. As an example of both the amount of available information and the insufficiency of naïve keyword search, the name of the protein *p53* occurs in 53,528 PubMed articles, and while a researcher interested specifically in its role in *cancer* and its interacting partners might try the search “*p53 cancer interaction*” to narrow down the results, this query still yields 1,777 publications, enough for months of full-time reading [1]. Nonetheless, PubMed is a very widely used free service and is providing an invaluable service to the researchers around the world. In March 2007, PubMed served 82 million (statistics of Medline searches: http://www.nlm.nih.gov/bsd/medline_growth.html) query searches and the usage is ever increasing. A few commercial products are currently available that provide additional services, but they also rely on basic keyword search, with no real discovery or dynamic faceted search. Examples are OvidSP and Ingenuity Answers, both of which support book-marking as one means of keeping track of visited citations. Research tools such as EBIMed (EBIMed: http://www.ebi.ac.uk/Rebholz-srv/ebimed/index.jsp) [2] and AliBaba (AliBaba: http://alibaba.informatik.hu-berlin.de) [3] provide additional cross-referencing of entities to databases such as UniProt or to the GeneOntology. They also try to identify relations between entities, such as protein-protein interactions, functional protein annotations, or gene-disease associations. 

Search tools should provide dedicated and intuitive strategies that help to find relevant literature, starting with initial keyword searches and drilling down results via overviews enriched with autogenerated suggestions to refine queries. One of the first steps in biomedical text mining is to recognize named entities occurring in a text, such as genes and diseases. Named entity recognition (NER) is helpful to identify relevant documents, index a document collection, and facilitate information retrieval (IR) and semantic searches [4]. A step on top of NER is to normalize each entity to a base form (also called grounding and identification); the base form often is an identifier from an existing, relevant database; for instance, protein names could be mapped to UniProt IDs [5, 6]. Entity normalization (EN) is required to get rid of ambiguities such as homonyms, and map synonyms to one and the same concept. This further alleviates the tasks of indexing, IR, and search. Once named entities have been identified, systems aim to extract relationships between them from textual evidences; in the biomedical domain, these include gene-disease associations and protein-protein interactions. Such relations can then be made available for subsequent search in relational databases or used for constructing particular pathways and entire networks [7].

Information extraction (IE) [8–11] is the extraction of salient facts about prespecified types of events, entities [12], or relationships from free text. Information extraction from free text utilizes shallow-parsing techniques [13], part-of-speech tagging [14], noun and verb phrase chunking [15], predicate-subject and object relationships [13], and learned [8, 16, 17] or hand-build patterns [18] to automate the creation of specialized databases. Manual pattern engineering approaches employ shallow parsing with patterns to extract the interactions. In the system presented in [19], sentences are first tagged using a dictionary-based protein name identifier and then processed by a module which extracts interactions directly from complex and compound sentences using regular expressions based on part of speech tags. IE systems look for entities, relationships among those entities, or other specific facts within text documents. The success of information extraction depends on the performance of the various subtasks involved.

The Suiseki system of Blaschke et al. [20] also uses regular expressions, with probabilities that reflect the experimental accuracy of each pattern to extract interactions into predefined frame structures. Genies [21] utilizes a grammar-based natural language processing (NLP) engine for information extraction. Recently, it has been extended as GeneWays [22], which also provides a Web interface that allows users to search and submit papers of interest for analysis. The BioRAT system [23] uses manually engineered templates that combine lexical and semantic information to identify protein interactions. The GeneScene system [24] extracts interactions using frequent preposition-based templates. 

Over the last years, a focus has been on the extraction of protein-protein interactions in general, recently including extraction from full text articles, relevance ranking of extracted information, and other related aspects (see, for instance, the BioCreative community challenge [25]). The BioNLP'09 Shared Task concentrated on recognition of more fine-grained molecular events involving proteins and genes [26]. Both papers give overviews over the specific tasks and reference articles by participants.

One of the first efforts to extract information on bio-molecular events was proposed by Yakushiji et al. [27]. They implemented an argument structure extractor based on full sentence parses. A list of target verbs have specific argument structures assigned to each. Frame-based extraction then searches for filler of each slot required according to the particular arguments. On an small in-house corpus, they found that 75% of the errors can be attributed to erroneous parsing and another 7% to insufficient memory; both causes might have less impact on recent systems due to more accurate parsers and larger memory.

Ding et al. [28] studied the extraction of protein-protein interactions using the Link Grammar parser. After some manual sentence simplification to increase parsing efficiency, their system assumed an interaction whenever two proteins were connected via a link path; an adjustable threshold allowed to cut off too long paths. As they used the original version of Link Grammar, Ding et al. [28] argued that adaptations to the biomedical domain would enhance the performance.

An information extraction application analyzes texts and presents only the specific information from them that the user is interested in [29]. IE systems are knowledge intensive to build and are to varying degrees tied to particular domains and scenarios such as target schema. Almost all IE applications start with fixed target schema as a goal and are tuned to extract information from unstructured text that will fit the schema. In scenarios where target schema is unknown, open information extraction systems [30] like KnowItNow [31] and TextRunner [32] allow rules to be defined easily based on the extraction need. An hybrid application (IR + IE) that leverages the best of information retrieval (ability to relevant texts) and information extraction (analyze text and present only specific information user is interested in) would be ideal in cases when the target extraction schema is unknown. An iterative loop of IR and IE with user's feedback will be potentially useful. For this application, we will need main components of IE system (like parts-of-speech tagger, named entity taggers, shallow parsers) preprocesses the text before being indexed by a custom-built augmented index that helps retrieve queries of the type “Cities such as ProperNoun(Head(Noun Phrase)).” Cafarella and Etzioni [33] have done work in this direction to build a search engine for natural language and information extraction applications.

Exploratory search [34] is a topic that has grown from the fields of information retrieval and information seeking but has become more concerned with alternatives to the kind of search that has received the majority of focus (returning the most relevant documents to a Google-like keyword search). The research is motivated by questions like “what if the user does not know which keywords to use?” or “what if the user is not looking for a single answer?”. Consequently, research began to focus on defining the broader set of information behaviors in order to learn about situations when a user is—or feels—limited by having only the ability to perform a keyword search (source: http://en.wikipedia.org/wiki/Exploratory_search). Exploratory search can be defined as specialization of information exploration which represents the activities carried out by searchers who are either [35]:

1. unfamiliar with the domain of their goal (i.e., need to learn about the topic in order to understand how to achieve their goal);
2. unsure about the ways to achieve their goals (either the technology or the process); or even
3. unsure about their goals in the first place.

A faceted search system (or parametric search system) presents users with key value metadata that is used for query refinement [36]. By using facets (which are metadata or class labels for entities such as genes or diseases), users can easily combine the hierarchies in various ways to refine and drill down the results for a given query; they do not have to learn custom query syntax or to restart their search from scratch after each refinement. Studies have shown that users prefer faceted search interfaces because of their intuitiveness and ease of use [37]. Hearst [38] shares her experience, best practices, and design guidelines for faceted search interfaces, focusing on supporting flexible navigation, seamless integration with directed search, fluid alternation between refining and expanding, avoidance of empty results sets, and most importantly making users at ease by retaining a feeling of control and understanding of the entire search and navigation process. To improve web search for queries containing named entities [39], automatically identify the subject classes to which a named entity might refer to and select a set of appropriate facets for denoting the query.

Faceted search interfaces have made online shopping experiences richer and increased the accessibility of products by allowing users to search with general keywords and browse and refine the results until the desired sub-set is obtained (SIGIR'2006 Workshop on Faceted Search (CFP): http://sites.google.com/site/facetedsearch/). Faceted navigation delivers an experience of progressive query refinement or elaboration. Furthermore, it allows users to see the impact of each incremental choice in one facet on the choices in other facets. Faceted search combines faceted navigation with text search, allowing users to access (semi) structured content, thereby providing support for discovery and exploratory search, areas where conventional search falls short [40].

## 2. Approach

 In an age of ever increasing published research documents (available in search-able textual form) containing amounts of valuable information and knowledge that are vital to further research and understanding, it becomes imperative to build tools and systems that enable easier and quick access to right information the user is seeking for, and this has already become an information overload problem in different domains. Information Extraction (IE) systems provide an structured output by extracting nuggets of information from these text document collections, for a defined schema. The output schema can vary from simple pairwise relations to a complex, nested multiple events.

Faceted search and navigation is an efficient way to browse and search over a structured data/document collection, where the user is concerned about the completeness of the search, not just top ranked results. Faceted search system needs structured input documents, and IE systems extract structured information from text documents. By combining these two paradigms, we are able to provide faceted search and navigation over unstructured text documents, and, with this fusion, we are also able to leverage real utility of information extraction, that is, finding hidden relationships as the user goes through a search process, and to help refine the query to more satisfying and relevant level, all while keeping user feel incontrol of the whole search process.

**Figure 1:**
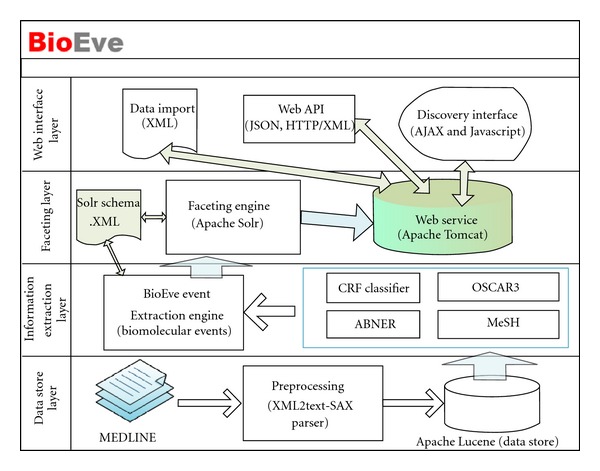
BioEve search framework architecture.

We developed BioEve Search (http://www.bioeve.org/) framework to provide fast and scalable search service, where users can quickly refine their queries and drill down to the articles they are looking for in a matter of seconds, corresponding to a few number of clicks. The system helps identify hidden relationships between entities (like drugs, diseases, and genes), by highlighting them using a tag cloud to give a quick visualization for efficient navigation. In order to have sufficient abstraction between various modules (and technologies used) in this system, we have divided this framework into four different layers (refer to Figure 1) and they are (a) Data Store layer, (b) Information Extraction layer, (c) Faceting layer, and (d) Web Interface layer. Next sections explain each layer of this framework in more details.

### 2.1. Data Store Layer

 The Data Store layer preprocesses and stores the documents in an indexed data store to make them efficiently accessible to the modules of upper layer (information extraction layer). Format conversion is needed sometimes (from ASCII to UTF-8 or vice versa), or XML documents need to be converted to text documents before being passed to next module. After the documents are in the required format and cleansed, they are stored in a indexed data store for efficient and fast access to either individual documents or the whole collections. The data store can be implemented using an Indexer service like (Apache Lucene (Lucene: http://lucene.apache.org/) or any database like MySQL). The Medline dataset is available as zipped XML files that needed XML2 text conversion, after which we could ingest them into an indexer, Apache Lucene in our case. Such an indexer allows for faster access and keyword-based text search to select a particular subset of abstracts for further processing. 

### 2.2. Information Extraction Layer

 For recognizing different gene/protein names, DNA, RNA, cell line, and cell types, we leveraged ABNER [41], A Biomedical Named Entity Recognizer. We used OSCAR3 (Oscar3: http://sourceforge.net/projects/oscar3-chem/) (Open Source Chemistry Analysis Routines) to identify chemical names and chemical structures. To annotate disease names, symptoms, and causes, we used a subset of the Medical Subject Heading (MeSH) dataset (MeSH: http://www.nlm.nih.gov/mesh/).

#### 2.2.1. Annotating Biomolecular Events in the Text

 A first step towards bio-event extraction is to identify phrases in biomedical text which indicate the presence of an event. The labeled phrases are classified further into nine event types (based on the Genia corpus (BioNLP'09 Shared Task 1: http://www.nactem.ac.uk/tsujii/GENIA/SharedTask/)). The aim of marking such interesting phrases is to avoid looking at the entire text to find participants, as deep parsing of sentences can be a computationally expensive process, especially for the large volumes of text. We intend to mark phrases in biomedical text, which could contain a potential event, to serve as a starting point for extraction of event participants.  Section 6.1 gives more details about our experimentations with classification and annotation of biomedical entities. 

All the classification and annotation were done *offline* before the annotated articles are indexed for the search as once an article is classified and annotated with different entity types, it does not need to be processed again for each search query. This step can be done preindexing and as a batch process.

### 2.3. Faceting Layer

#### 2.3.1. Faceting Engine

 To provide faceted classification and navigation over these categories (facets), many off-the-shelf systems are available such as in academia; Flamenco project (Flamenco: http://flamenco.berkeley.edu/) (from University of California Berkeley) and mspace (mspace: http://mspace.fm/) (University of Southampton) and in enterprise area; Apache Solr (Apache Solr: http://lucene.apache.org/solr/) and Endeca (Endeca: http://www.endeca.com/). We used the Apache Solr library for faceted search, which also provides an enterprise quality full-text search.

#### 2.3.2. Shared Schema between IE Layer and Faceting Layer

**Scheme 1:**
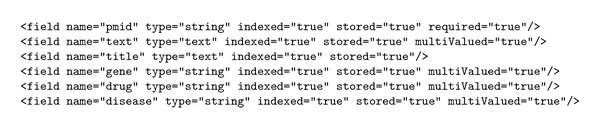


 In order to facilitate indexing and faceting over the extracted semi-structured text articles, both IE layer and faceting layer needs to share a common schema. A sample of shared schema used for enabling interaction between these layers is shown in Scheme 1.

### 2.4. Web Interface Layer

 With the advent of Web 2.0 technologies, web-based interfaces have undergone delightful improvements and now provide rich dynamic experiences. Key component in this layer is a user interface that connects the user with the web service from the faceting layer and provides features that allow search, selection of facet/values, refinement, query restart, and dynamic display of a result set as user interacts and navigates. It also provides the bulk import of data for further analysis of the faceting/extraction. 

The web interface provides following features for interactive search and navigation. The interface presents a number of entities types (on the left panel) along with the specific instances/values, from previous search results, and the current query. Users can choose any of the highlighted values of these entity types to interactively refine the query (add new values/remove any value from the list with just one click) and thereby drill down to the relevant articles quickly without actually reading the entire abstracts. Users can easily remove any of the previous search terms, thus widening the current search. We implemented the BioEve user interface using AJAX (AJAX: http://evolvingweb.github.com/ajax-solr/), Javascript, and JSON to provide rich dynamic experience. The web interface runs on an Apache Tomcat server. Next section explains about navigation aspect of the user interface.

## 3. User Interface: A Navigation Guide

**Figure 2:**
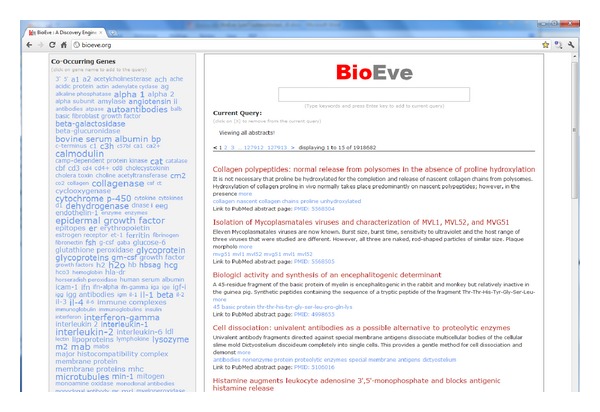
A sample screen shot of the main search screen. Left panel shows clickable top relevant entities, which if selected refines the query and results dynamically. User can deselect any of the previously selected entities to refine query more, and the results are updated dynamically to reflect the current selected list of entities.

Search interface is divided into left and right panels, see Figure 2, basically displaying enriched keywords and results, respectively.

*Left panel*: it offers suggestions and insights (based on cooccurrence frequency with the query terms) for different entities types, such as genes, diseases, and drugs/chemicals.

- Left panel shows navigation/refinement categories (genes, diseases, and drugs); users can click on any of the entity names (in light blue) to refine the search. By clicking on an entity, the user adds that entity to the search and the results on the right panel are refreshed on the y to reflect the refined results.
- Users can add or remove any number of refinements to the current search query until they reach the desired results set (shown in the right panel).

*Right panel*: it shows the user's current search results and is automatically refreshed based on user's refinement and navigation choices on the left panel.

- The top of the panel shows users current query terms and navigation so far. Here, users can also deselect any of the previously selected entities or even all of them by single click on “remove all.” By deselecting any entities, user is essentially expanding the search and the results in the right panel are refreshed *on the fly* to remaining query entities to offer a dynamic navigation experience.
- Abstracts results on this panel show “title” of the abstract (in light red), full abstract text (in black, if abstract text is available).
- Below the full abstract text, the list of entities mentioned in that abstracts (in light blue) is shown. These entities names are clickable and will start a new search for that entity name, with a single click.
- A direct URL is also provided to the abstract page on http://pubmed.gov in case the user wants to access additional information such as authors, publication type, or links to a full-text article.

## 4. Interactive Search and Navigation: A Walk through and behind the Scenes

Let us start an example search process, say with the query “cholesterol” and the paragraph titled “behind-the-scenes” gives details of the computational process behind the action.

(1) The autocomplete feature helps in completion of the name while typing if the word is previously mentioned in the literature, which is the case here with “cholesterol.”

*Behind-the-scenes:* as user starts typing, the query is tokenized (in case of multiple words) and search is made to retrieve word matches (and not the result rows yet) using the beginning with the characters user has already typed, and this loop continues. Technologies at play are jQuery, AJAX, and faceting feature of Apache Solr. Once the query is submitted by the user, the results rows also contain the annotated entity names and these are used to generate tag clouds, using the faceting classification entity frequency count. 

**Figure 3:**
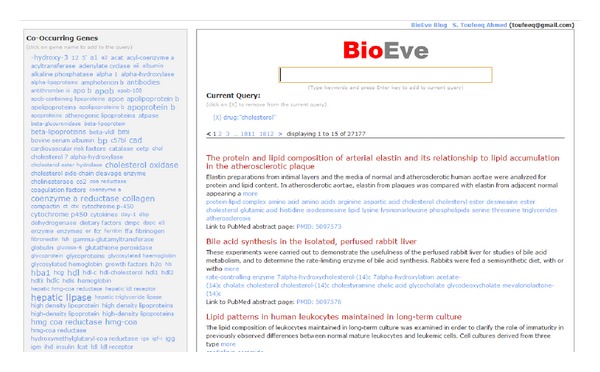
A sample result set with the query “cholesterol.”

The search results in 27177 articles hits (Figure 3). Those are a lot of articles to read. How about narrowing down these results with some insights given by BioEve Search?

**Figure 4:**
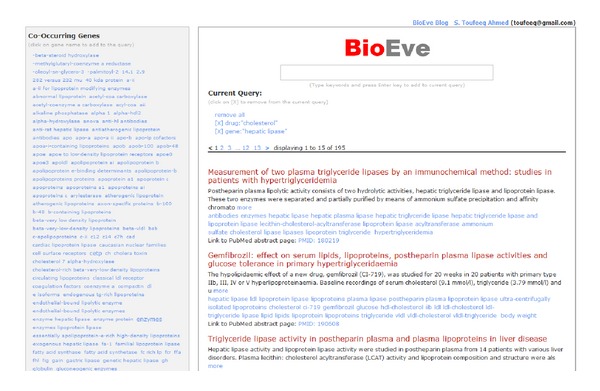
“Hepatic-lipase” selected.

(2) In left panel, “hepatic lipase” is highlighted; let us click on that as it shows some important relationship between “cholesterol” and “hepatic lipase.” The search results are now narrowed down to 195 articles from 27177 (Figure 4). That is still a lot of articles to read this afternoon, how about some insights on diseases. 

*Behind-the-scenes:* once user click on a highlighted entity name in tag cloud, this term (*gene: “hepatic lipase”*) is added to the search filter and the whole search process and tag could be generated again for the new query. 

**Figure 5:**
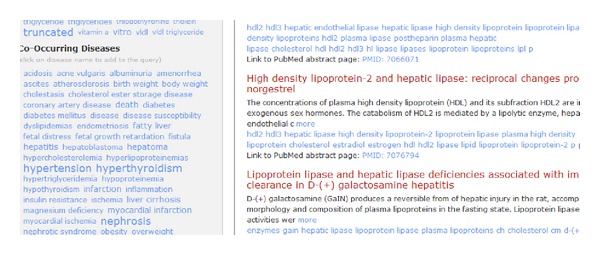
“Hyperthyroidism” highlighted.

You can see disease “hyperthyroidism” highlighted in Figure 5.

**Figure 6:**
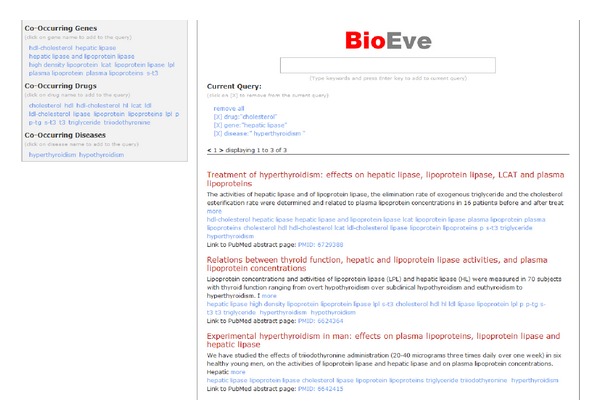
Final refined search results.

(3) Selecting “hyperthyroidism” drills results down to 3, as can be seen in Figure 6.

The top result is about “Treatment of hyperthyroidism: effects on hepatic lipase, lipoprotein lipase, LCAT and plasma lipoproteins”. With few clicks user can refine search results to more relevant articles.

## 5. Initial User Reviews and Feedback

We asked three life science researchers to review and provide feedback on ease of search and novelty of the system, and shown below is their feedback (paraphrased). Their names and other details are removed for privacy purposes. 

### 5.1. Researcher One, P.h.D, Research Fellow, Microbiology, University of California, Berkeley

“ I am impressed by ease of its use.” “When I have the confidence that BioEve is indexing all the data without missing any critical article, *I will be compelled to use this search tool.* I believe a finished product will be immensely useful and could become a popular tool for life science researchers.”

### 5.2. Researcher Two, P.h.D, Investigator and Head, Molecular Genetics Laboratory

“*You have a powerful search.* Synchronize this with MEDLINE. Connect with more databases, OMIM, Entrez Gene …. You can get cell line database from ATCC.org.”

### 5.3. Researcher Three, P.h.D, Postdoc Researcher, Faculty of Kinesiology, University of Calgary

“I particularly like the idea of having larger fonts for the more relevant terms highlighting what is researched more often.”

## 6. Methods

### 6.1. Information Extraction: Annotating Sentences with Biomolecular Event Types

 The first step towards bioevent extraction is to identify phrases in biomedical text which indicate the presence of an event. The aim of marking such interesting phrases is to avoid looking at the entire text to find participants. We intend to mark phrases in biomedical text, which could contain a potential event, to serve as a starting point for extraction of event participants. We experimented with well-known classification approaches, from a naïve Bayes classifier to the more sophisticated machine classification algorithms Expectation Maximization, Maximum Entropy, and Conditional Random Fields. Overview of different classifiers applied at different levels of granularity and the features used by these classifiers is shown in Table 1.

 For naïve Bayes classifier implementation, we utilized WEKA (WEKA: http://www.cs.waikato.ac.nz/ml/weka/) library, a collection of machine learning algorithms for data mining tasks, for identifying single label per sentence approach. WEKA does not support multiple labels for the same instance. Hence, we had to include a tradeoff here by including the first encountered label in the case where the instance had multiple labels. For Expectation Maximization (EM) and Maximum Entropy (MaxEnt) algorithms, we used classification algorithms from MALLET library (MALLET: http://mallet.cs.umass.edu/index.php). Biomedical abstracts are split into sentences. For training purposes, plain text sentences are transformed into training instances as required by MALLET.

#### 6.1.1. Feature Selection for Naïve Bayes, EM, and MaxEnt Classifiers

 For the feature sets mentioned below, we used the TF-IDF representation. Each vector was normalized based on vector length. Also, to avoid variations, words/phrases were converted to lowercase. Based on WEKA library token delimiters, features were filtered to include those which had an alphabet as a prefix, using regular expressions. For example, features like −300 bp were filtered out, but features like *p*55, which is a protein name, were retained. We experimented with the list of features described below, to understand how well each feature suits the corpus under consideration.

- Bag-of-words model: this model classified sentences based on word distribution.
- Bag-of-words with gene names boosted: the idea was to give more importance to words, which clearly demarcate event types. To start with, we included gene names provided in the training data. Next, we used the ABNER (ABNER: http://pages.cs.wisc.edu/~bsettles/abner/), a gene name tagger, to tag gene names, apart from the ones already provided to us. We boosted weights for renamed feature “protein”, by 2.0.
- Bag-of-words with event trigger words boosted: we separately tried boosting event trigger words. The list of trigger words was obtained from training data. This list was cleaned to remove stop words. Trigger words were ordered in terms of their frequency of occurrence with respect to an event type, to capture trigger words which are most discriminative.
- Bag-of-words with gene names and event trigger words boosted: the final approach was to boost both gene names and trigger words together. Theoretically, this approach was expected to do better than previous two feature sets discussed. Combination of discriminative approach of trigger words and gene name boosting was expected to train the classifier better.

#### 6.1.2. Evaluation of Sentence Level Classification Using Naïve Bayes Classifier

 This approach assigns a single label to each sentence. For evaluation purposes, the classifier is tested against GENIA development data. For every sentence, evaluator process checks if the event type predicted is the most likely event in that sentence. In case a sentence has more than one event with equal occurrence frequency, classifier predicted label is compared with all these candidate event types. The intent of this approach was to just understand the features suitable for this corpus. Classifier evaluated was NaiveBayesMultinomial classifier from Weka (http://www.cs.waikato.ac.nz/ml/weka/) library, which is a collection of machine learning algorithms for data mining tasks.  Table 2 shows precision results for NBC classifier with different feature sets for single label per sentence classification.

### 6.2. Conditional Random Fields Based Classifier

 Conditional Random fields (CRFs) are undirected statistical graphical models, a special case of which is a linear chain that corresponds to a conditionally trained finite-state machine [41]. CRFs in particular have been shown to be useful in parts-of-speech tagging [42] and shallow parsing [42]. We customized ABNER which is based on MALLET, to suit our needs. ABNER employs a set of orthographic and semantic features. 

#### 6.2.1. Feature Selection for CRF Classifier

The default model included the training vocabulary (provided as part of the BIONLP-NLPBA 2004 shared task) in the form of 17 orthographic features based on regular expressions [41]. These include upper case letters (initial upper case letter, all upper case letters, mix of upper and lower case letters), digits (special expressions for single and double digits, natural numbers, and real numbers), hyphen (special expressions for hyphens appearing at the beginning and end of a phrase), other punctuation marks, Roman and Greek words, and 3-gram and 4-gram suffixes and prefixes. ABNER uses semantic features that are provided in the form of hand-prepared (Greek letters, amino acids, chemical elements, known viruses, abbreviations of all these) and database-referenced lexicons (genes, chromosome locations, proteins, and cell lines).

### 6.3. Evaluation of Sentence Classification Approaches

 The framework is designed for large-scale extraction of molecular events from biomedical texts. To assess its performance, we evaluated the underlying components on the GENIA event dataset made available as part of BioNLP'09 Shared Task [26]. This data consists of three different sets: the training set consists of 800 PubMed abstracts (with 7,499 sentences), the development set has 150 abstracts (1,450 sentences), and the test set has 260 abstracts (2,447 sentences). We used the development set for parameter optimization and fine tuning and evaluated the final system on the test set. Employed classifiers were evaluated based on precision and recall. Precision indicates the correctness of the system, by measuring number of samples correctly classified in comparison to the total number of classified sentences. Recall indicates the completeness of the system, by calculating the number of results which actually belong to the expected set of results. Sentence level single label classification and sentence level multilabel classification approaches were evaluated based on how well the classifier labels a given sentence from a test set with one of the nine class labels. Phrase level classification using CRF model was evaluated based on how well the model tags trigger phrases. Evaluating this approach involved measuring the extent to which the model identifies that a phrase is a trigger phrase and how well it classifies a tagged trigger phrase under one of the nine predefined event types. Retrieved trigger phrases refer to the ones which are identified and classified by the CRF sequence tagger. Relevant trigger phrases are the ones which are expected to be tagged by the model. Retrieved and relevant trigger words refer to the tags which are expected to be classified and which are actually classified by the CRF model. All the classifiers are trained using BioNLP shared task training data and tested against BioNLP shared task development abstracts.

We compare the above three approaches for classification in Table 3. CRF has a good tradeoff as compared to Maximum Entropy classifier results. As compared to multiple labels, sentence level classifiers, it performs better in terms of having a considerably good accuracy for most of the event types with a good recall. It not only predicts the event types present in the sentence, but also localizes the trigger phrases. There are some entries where ME seems to perform better than CRF; for example, in case of *positive regulation*, where the precision is as high as 75%. However, in this case, the recall is very low (25%). The reason noticed (in training examples) was that, most of the true example sentences of positive regulation or negative regulation class type were misclassified as either phosphorylation or gene expression. The F1-score for CRF indicates that, as compared to the other approaches, CRF predicts 80% of the relevant tags, and, among these predicted tags, 68% of them are correct.

#### 6.3.1. Evaluation of Phrase Level Labeling

Evaluation of this approach was focused more on the overlap of phrases between the GENIA annotated development and CRF tagged labels. The reason being for each abstract in the GENIA corpus, there is generally a set of biomedical entities present in it. For the shared task, only a subset of these entities was considered in the annotations, and accordingly only events concerning these annotated entities were extracted. However, based on the observation of the corpus, there was a probable chance of other events involving entities not selected for the annotations. So we focused on the coverage, where both the GENIA annotations and CRF annotations agree upon. CRF performance was evaluated on two fronts in terms of this overlap.

- *Exact boundary matching*: this involves exact label matching and exact trigger phrase match.
- *Soft boundary matching*: this involves exact label matching and partial trigger phrase match, allowing 1-word window on either side of the actual trigger phrase.

Checking of the above constraints was a combination of template matching and manually filtering of abstracts.  Table 4 gives an estimate of the coverage. Soft boundary matching increases the coverage by around 3%. Table 3 gives the overall evaluation of CRF with respect to GENIA corpus. With regards to the CRF results, accuracy for *positive regulation* is comparatively low. Also, the test instances for *positive regulation* were more than any other event type. So this reduced average precision to some extent.

A detailed analysis of the results showed that around 3% tags were labeled incorrectly in terms of the event type. There were some cases where it was not certain whether an event should be marked as *regulation* or *positive regulation*. Some examples include “the expression of LAL-mRNA,” where “LAL-mRNA” refers to a gene. As per examples seen in the training data, the template of the form “expression of <gene name>” generally indicates presence of a *Gene expression* event. Hence, more analysis may be need to exactly filter out such annotations as true negatives or deliberately induced false positives.

## 7. Discussion and Conclusions

PubMed is one of the most well known and used citation indexes for the Life Sciences. It provides basic keyword searches and benefits largely from a hierarchically organized set of indexing terms, MeSH, that are semi-automatically assigned to each article. PubMed also enables quick searches for related publications given one or more articles deemed relevant by the user. Some research tools provide additional cross-referencing of entities to databases such as UniProt or to the GeneOntology. They also try to identify relations between entities of the same or different types, such as protein-protein interactions, functional protein annotations, or gene-disease associations. GoPubMed [43] guides users in their everyday searches by mapping articles to concept hierarchies, such as the Gene Ontology and MeSH. For each concept found in abstracts returned by the initial user query, GoPubMed computes a rank based on occurrences of that concept. Thus, users can quickly grasp which terms occur frequently, providing clues for relevant topics and relations, and refine subsequent queries by focusing on particular concepts, discarding others.

In this paper, we presented BioEve Search framework, which can help identify important relationships between entities such as drugs, diseases, and genes by highlights them during the search process. Thereby, allowing the researcher not only to navigate the literature, but also to see entities and the relations they are involved in immediately, without having to fully read the article. Nonetheless, we envision future extensions to provide a more complete and mainstream service and here are few of these next steps.

Keeping the search index up-to-date and complete: we are adding a synchronization module that will frequently check with Medline for supplement articles as they are published; these will typically be in the range of 2500–4500 new articles per day. Frequent synchronization is necessary to keep BioEve abreast with Medline collection and give users the access to the most recent articles.

Normalizing and grounding of entity names: as the same gene/protein can be referred by various names and symbols (e.g., the TRK-fused gene is also known as TF6; TRKT3; FLJ36137; TFG), a user searching for any of these names should find results mentioning any of the others. Removal of duplicates and cleanup of nonbiomedical vocabulary that occurs in the entity tag clouds will further improve navigation and search results.

Cross-referencing with biomedical databases: we want to cross-reference terms indexed with biological databases. For example, each occurrence of a gene could be linked to EntrezGene and OMIM; cell lines can be linked and enriched with ATCC.org's cell line database; we want to cross-reference disease names with UMLS and MeSH to provide access to ontological information. To perform this task of entity normalization, we have previously developed Gnat [6], which handles gene names. Further entity classes that exhibit relatively high term ambiguity with other classes or within themselves are diseases, drugs, species, and GeneOntology terms (“Neurofibromatosis 2” can refer to the disease or gene).

## Figures and Tables

**Table 1:** Classification approaches used: Naïve Bayes classifier (NBC), NBC + Expectation Maximization (EM), Maximum Entropy (MaxEnt), Conditional Random Fields (CRFs).

Granularity	Features	Classifier
Single label,	Bag-of-words (BOW)	NBC
Sentence level	BOW + gene names boosted	
	BOW + trigger words boosted	
	BOW + gene names and trigger	
	words boosted	

Multiple labels	BOW	NBC +
		EM
Sentence level		MaxEnt

Event trigger	BOW +	CRFs
phrase labeling	3-gram and 4-gram	
	prefixes and suffixes +	
	orthographic features +	
	trigger phrase dictionary	

**Table 2:** Single label, sentence level results.

Classifier	Feature set	Precision
	Bag-of-words	62.39%
	Bag-of-words + gene name boosting	50.00%
NBC	Bag-of-words + trigger word boosting	49.92%
	Bag-of-words + trigger word boosting +	49.77%
	Gene name boosting	
	Bag-of-POS tagged words	43.30%

**Table 3:** Summary of classification approaches: test instances (marked events) for each class type in test dataset. Precision, recall, and F1-score in percentage. Compared to NB + EM and CRF, Maximum Entropy based classifier had better average precision, but CRF has best recall and good precision, giving it best F-Measure of the three well-known classifiers.

Event type	Test instances	NB + EM			MaxEnt			CRF		
	Total: 942	P	R	F1	P	R	F1	P	R	F1
Phosphorylation	38	62	42	50	97	73	83	80	83	81
Protein catabolism	17	60	47	53	97	73	83	85	86	85
Gene expression	200	60	41	49	88	58	70	75	81	78
Localization	39	39	47	43	61	69	65	67	79	72
Transcription	60	24	52	33	49	80	61	57	78	66
Binding	153	56	63	59	65	62	63	65	81	72
Regulation	90	47	69	55	52	67	58	62	73	67
Positive regulation	220	70	27	39	75	25	38	55	74	63
Negative regulation	125	42	46	44	54	38	45	68	82	74

Average		51	48	47	**71 **	61	63	68	** 80 **	** 73 **

**Table 4:** CRF sequence labeling results.

Type of evaluation	Coverage %
Exact boundary matching	79%
Soft boundary matching	82%
